# Cytotoxic Illudalane Sesquiterpenes from the Wood-Decay Fungus *Granulobasidium vellereum* (Ellis & Cragin) Jülich

**DOI:** 10.3390/molecules190914195

**Published:** 2014-09-09

**Authors:** Christina L. Nord, Audrius Menkis, Anders Broberg

**Affiliations:** 1 Department of Chemistry and Biotechnology, Uppsala BioCenter, Swedish University of Agricultural Sciences, P.O. Box 7015, Uppsala SE-750 07, Sweden; E-Mail: Christina.Nord@slu.se; 2 Department of Forest Mycology and Plant Pathology, Uppsala BioCenter, Swedish University of Agricultural Sciences, P.O. Box 7026, Uppsala SE-750 07, Sweden; E-Mail: Audrius.Menkis@slu.se

**Keywords:** *Granulobasidium vellereum*, sesquiterpenes, illudalane, cytotoxic

## Abstract

Seven illudalane sesquiterpenes were obtained from the wood decomposing fungus *Granulobasidium vellereum*: granuloinden A, granuloinden B and dihydrogranuloinden, along with the previously known compounds radulactone, pterosin M, echinolactone A and D. Granuloinden B showed potent cytotoxic activity against the Huh7 and MT4 tumor cell lines (CC_50_ values of 6.7 and 0.15 µM, respectively), whereas granuloinden A and dihydrogranuloinden had no or moderate activity.

## 1. Introduction

Illudalane sesquiterpenes have primarily been isolated from ferns of the family *Pteridaceae* [1] and fungi of the phylum Basidiomycota [2,3,4]. Biological activities that have been ascribed to the illudalane type sesquiterpenes include among others, cytotoxicity [5,6,7]. The illudalanes produced by ferns are sometimes glycosylated, which has been reported to increase the cytotoxicity of the compounds, though there are examples of some potent cytotoxins among the non-glycosylated illudalanes [6,7].

The wood decomposing fungus *Granulobasidium vellereum* (Ellis & Cragin) Jülich (syn. *Hypochnicium vellereum* (Ellis & Cragin) Parmasto) has previously been found to produce protoilludane sesquiterpenes with moderate biological activity [8,9]. Here we describe the isolation and characterization of seven illudalane sesquiterpenes produced by *G. vellereum*, of which three have not previously been described, and among which one showed potent cytotoxic activity.

## 2. Results and Discussion

**Figure 1:**
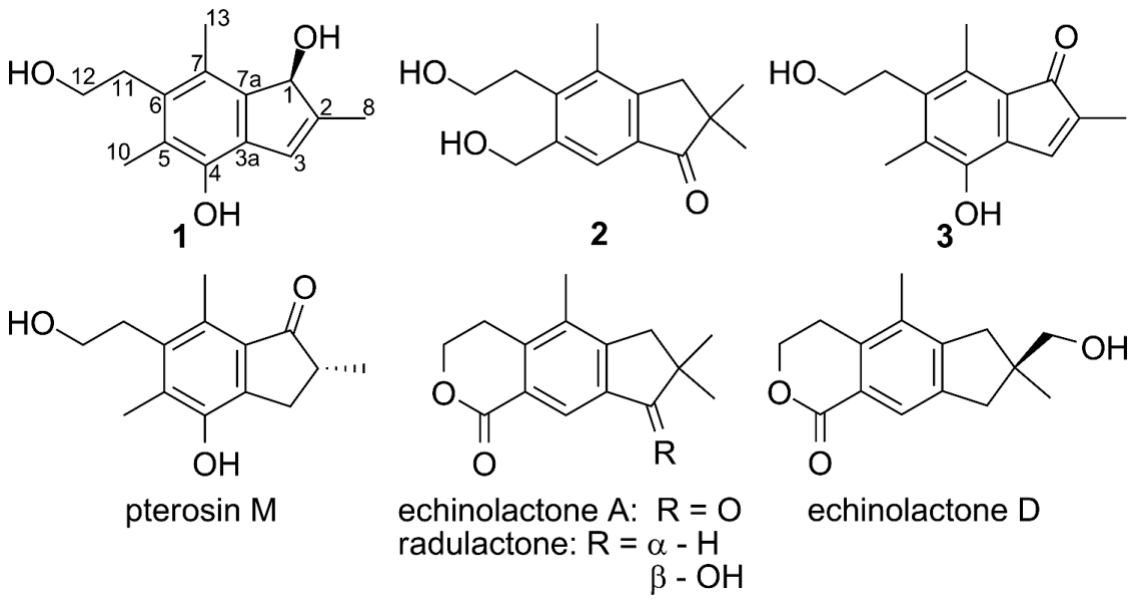
Structures of compounds **1**–**3**, pterosin M, radulactone, echinolactone A and D.

Seven illudalane sesquiterpenes were isolated from the saprotrophic fungus *G. vellereum* using solid-phase extraction (SPE) and preparative HPLC: granuloinden A (**1)**, dihydrogranuloinden (**2**), granuloinden B (**3**), radulactone, pterosin M, echinolactone A and D (Figure 1). The structures were determined with spectroscopic techniques and the cytotoxic effects of **1**–**3** were evaluated against the Huh7 and MT4 cancer cell lines. 

The determinations of the structures of radulactone, pterosin M and echinolactone A and D were based on the comparison of their MS, NMR and polarimetric data to those reported in the literature [2,3,4,10,11]. Pterosin M has been isolated as the aglycon of pteroside M, but has not previously been reported as a natural product [10], whereas radulactone had been isolated from the fungus *Radulomyces confluens* [2] and echinolactone A and D from *Echinodontium japonicum* [3,4].

Compound **1** was obtained as a light yellow crystalline compound and was the most abundant of the seven identified illudalanes, with a maximum yield of 77.3 mg/L filtrate. The molecular composition was C_14_H_18_O_3_ according to HRMS, indicating a degree of unsaturation of six and since the ^13^C-NMR spectrum displayed the presence of eight sp^2^ carbons, the structure must be bicyclic (Table 1).

**Table 1:** ^1^H- (600 MHz) and ^13^C-NMR (100 MHz) data for compounds **1**–**3** (MeOH*-d*_4_, 30°C).

pos.	1		2		3	
	δ*_C_*, mult	δ*_H_* ( *J* in Hz)	δ_*C*,_ mult	δ*_H_* (*J* in Hz)	δ_*C*,_ mult.	δ*_H_* (*J* in Hz)
1	73.5, CH	4.81, m	43.3, CH_2_	2.94, s	201.8,	C
2	146.5, C		46.7		134.4, C	
3	123.9, CH	6.47, dd (1.7, 2.7)	214.2,	C	140.8, CH	7.38, q (1.8)
3a	128.9, C		134.1, C		127.5, C	
4	146.3, C		122.4, CH,	7.61, s	147.6, C	
5	125.8, C		141.6, C,		134.4, C	
6	134.0, C		144.6, C,		138.8, C	
7	126.5, C		136.3, C,		131.2, C	
7a	143.1, C		153.0, C,		127.2, C	
8	13.9, CH_3_	2.01, dd (0.9, 1.7)	25.6, CH_3_	1.20, s	10.0, CH_3_	1.76, d (1.8)
9			25.6, CH_3_	1.20, s		
10	12.3, CH_3_	2.20, s	63.5, CH_2_	4.73, s	12.9, CH_3_	2.19, s
11	34.0, CH_2_	2.91, m	33.6, CH_2_	3.10, t (7.4)	33.4, CH_2_	2.87, t (7.5)
12	62.2, CH_2_	3.55, m	62.1, CH_2_	3.72, t (7.4).	61.8, CH_2_	3.56, t (7.5)
13	14.9, CH_3_	2.35, s	14.7, CH_3_	2.35, s	12.8, CH_3_	2.42, s

**Figure 2:**
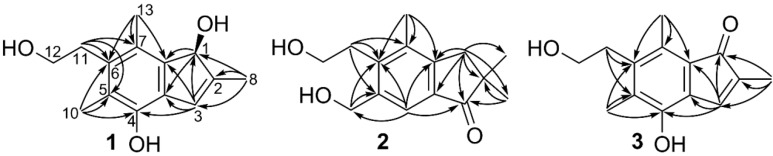
Diagnostic HMBC NMR correlations of compounds **1**–**3** used for structure determination. The HMBC data was obtained in MeOH-*d*_4_.

Through COSY and HSQC experiments a –CH_2_(11)-CH_2_OH(12) spin system was identified, and the NMR data further indicated the presence of three methyl groups and an isolated CHOR group. From methyl-8 an HMBC NMR correlation to the isolated CHOR group (C-1) as well as to two sp^2^ carbons (C-2 and C-3) were detected. H-3 correlated to C-1 and to three sp^2^ carbons C-3a, C-4 and C-7a. H-1 showed correlation to C-3a and C-7a together forming a cyclopentadiene moiety (Figure 2). C-7a correlated also to methyl-13, which in turn correlated to two additional sp^2^ carbons (C-6 and C-7). Methyl-10 correlated to C-4, C-5 and C-6, of which the latter also correlated to the H_2_-11, rendering in the proposed structure of compound **1**, which seems to be of sesquiterpenoid origin but has lost one carbon during the biosynthesis. Due to the indene structural backbone of the compound the name granuloinden A was suggested.

**Figure 3:**
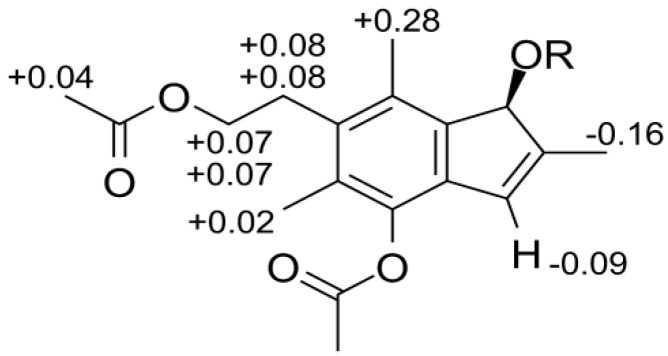
Chemical shift differences (in ppm) between the S-MTPA monoester of **1a ** and the R-MTPA monoester of **1a**. The data was obtained in acetone-*d_6_*.

Mosher’s test was preformed to be able to determine the configuration of the compound [12], but since compound **1** has three hydroxyl groups of which all might react with the chiral reagent α-methoxy(trifluoromethyl)phenylacetyl chloride (MTPA-Cl) used for the test, compound **1** was selectively acetylated on the hydroxyl groups on carbons 4 and 12 to produce compound **1a** (Figure S19). Treatment of **1a ** with MTPA-Cl yielded the S-MTPA and R-MTPA monoesters of **1a**, which respective ^1^H-NMR shifts were assigned (Table S1). From the comparison of the shift differences between the S- and R-MTPA monoesters of **1a** a 7*S* configuration was determined (Figure 3).

HRMS determined the molecular formula of compound **2** to be C_15_H_20_O_3_ and it had consequently a degree of unsaturation of six. According to the ^13^C-NMR spectrum the structure contained one carbonyl group and six other sp^2^ carbons (Table 1), leading to the conclusion that the compound must be bicyclic. From COSY and HSQC NMR data one -CH_2_(11)-CH_2_OH(12) spin system was identified. HMBC NMR showed correlations from the CH_2_ end of the spin system to three sp^2^ carbons (C-5, C-6 and C-7) (Figure 2). Methyl-13 had also HMBC correlations to C-6 and C-7 as well as an additional sp^2^ carbon (C-7a), the latter correlated in turn to an isolated CH_2_ group (H_2_-1). H_2_-1 had HMBC cross peaks to a quaternary carbon (C-2), to the sp^2^-carbon C-3a, to the geminal methyl groups 8 and 9 as well as to a carbonyl group (C-3), which in turn had a cross peak to a proton linked to a sp^2^ carbon (H-4). H-4 had HMBC correlations to C-6 and C-7a and to an isolated CH_2_OH group (C-10). The latter of which also had correlations to C-5 and C-6, thus concluding the structure elucidation of compound **2**, for which the name dihydrogranuloinden was proposed. 

Compound **3** was obtained as a strongly orange colored powder and had molecular formula of C_14_H_16_O_3_ according to HRMS analysis. The ^1^H- and ^13^C-NMR data displayed substantial similarities with those of compound **1** (Table 1) and from 2D NMR data it could be deduced that the only difference between the two was that C-1 had been oxidized to a carbonyl in compound **3 ** (Figure 2) and it was consequently named granuloinden B. Compound **3 **was isolated from the liquid culture but was also formed in small amounts by auto-oxidation of compound **1**, making it uncertain whether the formation of compound **3** actually is an enzymatic process or if it is formed as a degradation product of **1**.

**Figure 4:**
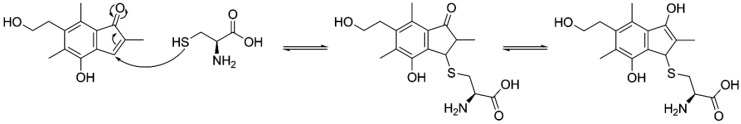
Suggested mechanism of the reaction between L-cysteine and compound **3**.

Compound **3** showed strong cytotoxic activity against both the Huh7 and MT4 cell lines, with CC_50_ values of 6.7 and 0.15 µM, respectively, whereas compounds **1** and **2** showed no cytotoxicity against Huh7 cells at concentrations up to 400 µM and only moderate cytotoxic activity against MT4 cells (Table 2). This result might be due to the fact that compound **3**, unlike compounds **1** and **2**, has an α,β-unsaturated carbonyl group which may facilitate a Michael type reaction. The cytotoxic effect of the known alkylating agents illudin M and S has been partially explained by invoking a similar mechanism, involving a Michael type addition of thiols in e.g. amino acids to an α,β-unsaturated carbonyl moiety [13,14]. To test if **3** would react with cysteine in a similar way as described for illudin M and S [14], compound **3** was dissolved in acetate buffer (pH 5.4, 50 mM) and reacted with a large excess of cysteine to show pseudo first order kinetics. Indeed formation of a product arising from a Michael type addition of cysteine to **3** occured rapidly (Figure 4), indicating that **3** may have a similar mechanism of action in the cell as illudin M and S.

**Table 2:** Cytotoxic activities of compounds **1**–**3** against the Huh7 and MT4 tumor cell lines.

Cell Line	CC_50_ (µM)		
	1	2	3
Huh7	>400	>400	6.7
MT4	55	180	0.15

## 3. Experimental Section

### 3.1. General Procedures

Preparative HPLC was performed on a Gilson 305/306 system, equipped with a Gilson 118 UV/VIS detector (254 nm). The NMR data were recorded at 303 K on a Bruker Avance III 600 MHz NMR spectrometer (5 mm QXI probe, 5 mm CryoProbe or a 5 mm SmartProbe) or on a Bruker DRX400 NMR spectrometer (5 mm QNP probe). The chemical shifts are reported relative to the residual solvent signal of MeOH-*d_4_* (δ_H_ 3.31; δ_C_ 49.00). HRMS data were obtained on a Bruker maXis Impact ESI UHR Q-TOF with Na formate (positive) as calibrant. The optical rotation was measured with a Perkin Elmer 341 polarimeter (λ 589 nm, path length 10.0 cm, 20 °C). The UV-data was recorded on a Hitachi U-2001 spectrophotometer and the melting point was measured on an Electrothermal 9100, using Vitrex S80 (1.4 × 80 mm) capillary tubes.

### 3.2. Fungal Cultivation

Isolation and identification of fungal culture of *G. vellereum* strain olrim243 is described in a previous study [8]. For the production of metabolites, *G. vellereum* was grown in 500 mL Erlenmeyer flasks each containing 250 mL of liquid Hagem medium [15]. Five agar plugs 0.5 × 0.5 cm in size with established fungal mycelia from an actively growing colony were aseptically inoculated in each flask and incubated on a rotary shaker at 120 rpm at room temperature (*ca.* 21 °C) for average periods of four weeks. After cultivation, cultures were filtered to obtain mycelium-free samples.

### 3.3. Bioassay Procedure

MT4 cells (T-cell line, a kind gift from Prof. Yamamoto, Yamaguchi University, Japan) were maintained in Roswell Park Memorial Institute (RPMI) medium supplemented with 10% heat inactivated fetal calf serum, penicillin (100 U/mL) and streptomycin (100 µg/mL). Huh7 cells (hepatocarcinoma cell line, ReBlikon GmbH, Schriesheim, Germany) were maintained in Dulbecco’s modified Eagle’s medium (D-MEM) supplemented with 10% heat inactivated fetal calf serum, penicillin (100 U/mL) and streptomycin (100 µg/mL). Briefly, cells were passaged into 96 well microplates (2 × 10^4^ cells/well) and the following day, test compounds in two-fold serial dilutions were added in DMSO. After six days the number of viable cells in each well was assessed by using a soluble formazan (XTT) assay [16] and the concentration causing 50% decrease in cell proliferation (CC_50_) was determined.

### 3.4. Isolation of Compounds from Liquid Cultures

The cell free filtrates of *G. vellereum* were extracted on 10-g SPE columns (50 mL filtrate per 1 g packing material; C18 (EC), International Sorbent Technology, Hengoed, UK). The columns were washed with water to remove the non-bonding materials before eluting the more lipofilic substances with aqueous 95% MeCN. The combined lipofilic fractions were dried in a vacuum centrifuge and redissolved in aqueous 40% MeCN before fractionation by preparative reversed-phase HPLC (linear gradient 10%–95% MeCN in water in 10 minutes, followed by a hold at 95% MeCN in 10 min, at 10 mL/min, Reprosil-Pur ODS-3, C_18_, 5 μm, 100 × 20 mm and guard column 30 × 20 mm, Dr Maisch GmbH, Ammerbuch, Germany). The fractionation was monitored by a UV-detector at 254 nm and 2 mL fractions were collected in deep-well plates.

The fractions containing crude compound **1** were pooled and chromatographed over Sephadex LH-20 (Pharmacia; 10 g; 30 × 1.5 cm) using aqueous 15% MeCN as eluent. The fractions containing compounds **2**, **3**, radulactone, pterosin M and echinolactone A and D were individually pooled and rechromatographed using preparative reversed-phase HPLC (same column as above) under isocratic conditions with 22% (**2**), 25% (pterosin M) and 30% (**3**, radulactone, echinolactone A and D) aq. MeCN, respectively, at 13.2 mL/min. The maximum yields of the compounds were 77.3 (**1**), 0.8 (**2**), 0.9 (**3**), 4.9 (radulactone), 0.4 (pterosin M), 0.4 (echinolactone A) and 1.7 (echinolactone D) mg/L filtrate of *G. vellereum*.

#### 3.4.1. Granuloinden A (**1**)

Compound **1** was obtained as a light yellow crystalline compound, m.p. 173–176 °C. [α]_D_ 65 (*c* 0.7 in MeOH); UV λ_max_ (MeOH) nm (log ε): 320, 274, 232, 208 (3.3, 3.4, 4.0, 4.1); NMR-data, see Table 1; HRMS *m/z* 257.1149 [M + Na]^+^ (calcd. for C_14_H_18_NaO_3_, 257.1148).

Formation of Compound **1a**

To a solution of compound **1** (8.3 mg, 0.04 mmol) in pyridine (500 µL), acetic anhydride (8.4 µL, 0.09 mmol) was added at 0 °C. The reaction was stirred for 24 h, after which the crude mixture was washed with 3 × 0.5 mL of water. The solvent was then evaporated and the crude product was redissolved in 50% aqueous MeCN, before being purified by preparative reversed-phase HPLC (aqueous 10%–95% MeCN 1–10 min and then a hold at 95% MeCN for 10 min; column as above, 10.0 mL/min) yielding 1.3 mg (12%) of the desired product. 

Formation of the (*S*)-MTPA Ester of Compound **1a**

To a solution of compound **1a** (0.62 mg, 0.0026 mmol) in pyridine-*d_5_* (500 µL) *R*-(‒)-MTPA-Cl (4.6 µL, 0.027 mmol) was added in r.t., and the reaction mixture was left stirring for 72 h. The solvent was then evaporated and the crude product was redissolved in 50% aqueous MeCN, before being purified by preparative reversed-phase HPLC (aqueous 10%–95% MeCN 1–10 min and then a hold at 95% MeCN for 10 min; column as above, 10.0 mL/min) yielding the desired product, after which the solvent was evaporated and the product redissolved in acetone-*d*_6_ and analysed by NMR.

Formation of the (*R*)-MTPA Ester of Compound **1a**

The same experimental procedure as above, with S-(+)-MTPA-Cl (4.3 µL, 0.025 mmol) and **1a** (0.58 mg, 0.0025 mmol) dissolved in pyridine-*d*_5_ (500 µL).

#### 3.4.2. Dihydrogranuloinden (**2**)

Compound **2** was obtained as a yellow oil. UV λ_max_ (MeOH) nm (log ε): 260, 214 (4.0, 4.3); NMR-data, see Table 1; HRMS *m/z* 249.1486 [M + H]^+^ (calcd. for C_15_H_21_O_3_, 249.1485).

#### 3.4.3. Granuloinden B (**3**)

Compound **3** was obtained as an orange powder. UV λ_max_ (MeOH) nm (log ε) 442, 366, 246, 206 (3.3, 3.6, 4.5, 4.5); NMR-data, see Table 1; HRMS *m/z* 233.1180 [M + H]^+^ (calcd. for C_14_H_17_O_3_, 233.1172).

#### 3.4.4. Reaction of **3** with Cysteine

Compound **3** (0.5 mgm, 2.2 µmol) was dissolved in acetate buffer (pH 5.4, 5 mL, 50 mM) and L-cysteine (4.6 mg, 38 µmol) was added. The reaction was monitored with LC-HRMS (aqueous 10%–90% MeCN, 10 min and then a hold at 90% for 10 min; Reprosil-Pur ODS-3, C_18_, 3.5 μm, 125 × 4.6 mm, Dr Maisch GmbH, Ammerbuch, Germany), which showed that reaction product was formed rapidly. The product was purified by preparative reversed-phase HPLC (aqueous 10%–90% MeCN, 10 min and then a hold at 90% MeCN for 10 min; column as above, 10.0 mL/min) and analyzed by NMR (methanol-*d*_4_). HRMS; *m/z* 354.1372 [M + H]^+^ (calcd. for C_17_H_24_NO_5_S, 354.1370).

## 4. Conclusions

The present study extends our knowledge of the secondary metabolites of *G. vellereum*, to also now include seven illudalane sesquiterpenes of which one, granuloindene B, showed potent cytotoxic activity against the Huh7 and MT4 tumor cell lines. The cytotoxic effect of granuloindene B is probably due to its chemical reactivity (Michael type addition) and not to enzymatic effects. It is possible that granuloindene B has a similar mechanism of action in the cell as the known alkylating agents illudin M and S.

## Supplementary Materials

Supplementary materials can be accessed at: http://www.mdpi.com/1420-3049/19/9/14195/s1..

## Supplementary Files

Supplementary File 1
