# Supplementary material for: Cytotoxic Illudalane Sesquiterpenes from the Wood-Decay Fungus Granulobasidium vellereum (Ellis & Cragin) Jülich
Source: Molecules. 2014 Sep 9;19(9):14195–203. doi: 10.3390/molecules190914195 (PMC6271737; doi:10.3390/molecules190914195)
Supplement: Supplementary File 1 [file molecules-19-14195-s001.pdf]

Supplementary Materials

Contents

Figure S1. ^{1}H-NMR (methanol-d_4, 600 MHz) spectrum of **1**. S2
Figure S2. ^{13}C-NMR (methanol-d_4, 100 MHz) spectrum of **1**. S2
Figure S3. ^{1}H-NMR (methanol-d_4, 600 MHz) spectrum of **2**. S3
Figure S4. ^{13}C-NMR (methanol-d_4, 100 MHz) spectrum of **2**. S3
Figure S5. ^{1}H-NMR (methanol-d_4, 600 MHz) spectrum of **3**. S4
Figure S6. ^{13}C-NMR (methanol-d_4, 100 MHz) spectrum of **3**. S4
Figure S7. ^{1}H-NMR (methanol-d_4, 600 MHz) spectrum of pterosin M. S5
Figure S8. ^{13}C-NMR (methanol-d_4, 100 MHz) spectrum of pterosin M. S5
Figure S9. ^{1}H-NMR (methanol-d_4, 600 MHz) spectrum of echinolactone D. S6
Figure S10. ^{13}C-NMR (methanol-d_4, 100 MHz) spectrum of echinolactone D. S6
Figure S11. ^{1}H-NMR (methanol-d_4, 600 MHz) spectrum of radulactone. S7
Figure S12. ^{13}C-NMR (methanol-d_4, 100 MHz) spectrum of radulactone. S7
Figure S13. ^{1}H-NMR (methanol-$d_{4,}$ 600 MHz) spectrum of echinolactone A. S8
Figure S14. ^{13}C-NMR (methanol-d_4, 100 MHz) spectrum of echinolactone D. S8
Figure S15. ^{1}H-NMR (acetone-d_6, 600 MHz) spectrum of **1a**. S9
Figure S16. ^{1}H-NMR (acetone-d_6, 600 MHz) spectrum of the (S)-MTPA ester of **1a**. S9
Figure S17. ^{1}H-NMR (acetone-d_6, 600 MHz) spectrum of the (R)-MTPA ester of **1a**. S10
Figure S18. ^{1}H-NMR (methanol-d_6, 600 MHz) spectrum of the reaction product of **3** and cysteine. ..S10
Figure S19. Structure of **1a**(R = OH) and the R- and S-MTPA monoesters of **1a**(R = R-MTPA and R = S-MTPA, respectively). S11
Table S1. ^{1}H-NMR data for compound **1a**and the R- and S-MTPA monoesters of **1a**(acetone-d_6, 30 °C, 600 MHz) S11

Figure S1. ^{1}H-NMR (methanol-d_4, 600 MHz) spectrum of **1**.

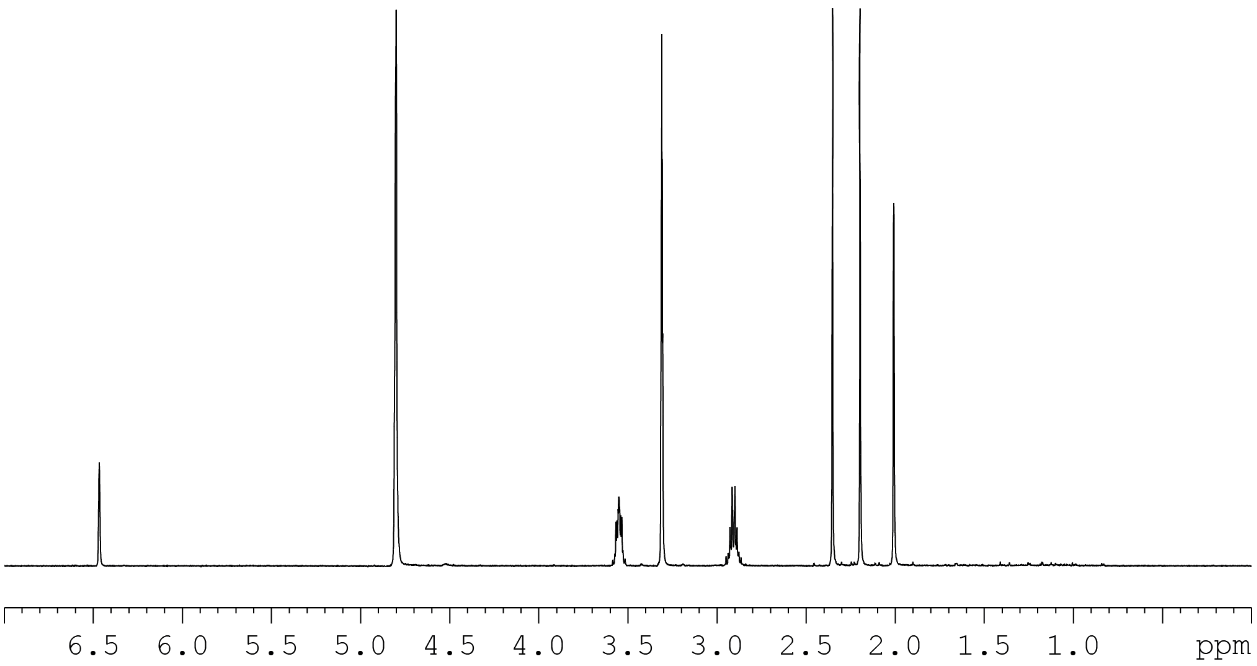

Figure S2. ^{13}C-NMR (methanol-d_4, 100 MHz) spectrum of **1**.

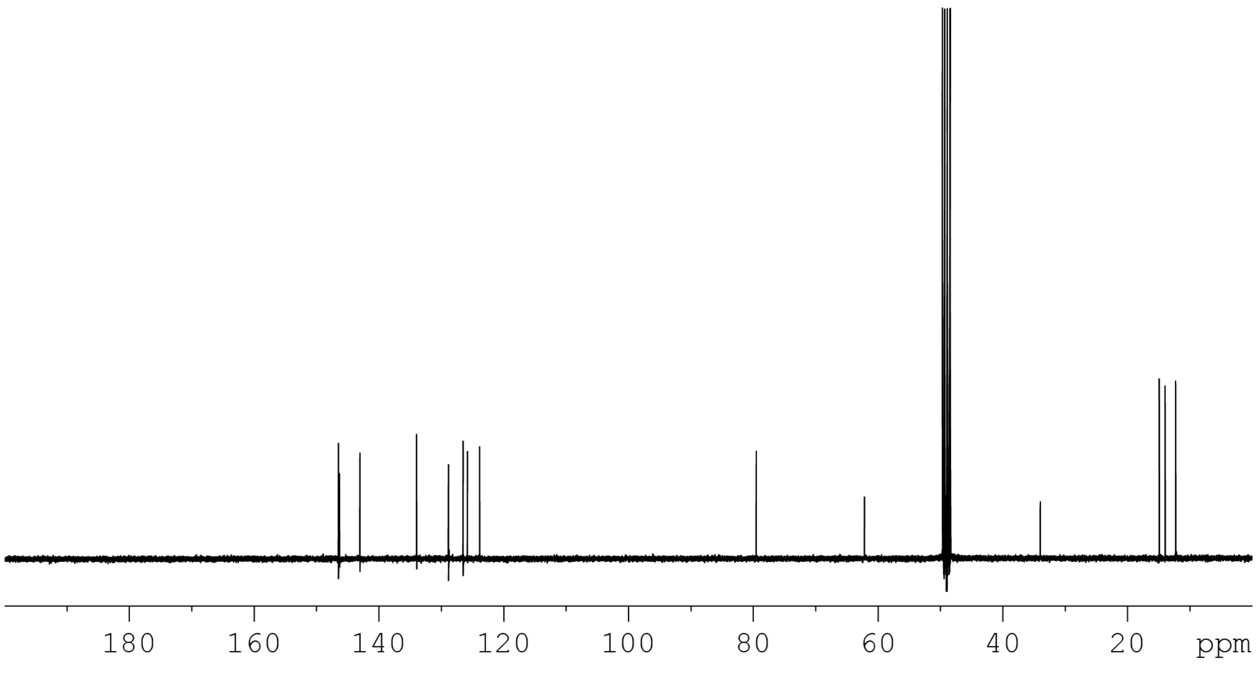

Figure S3. ^{1}H-NMR (methanol-d_4, 600 MHz) spectrum of **2**.

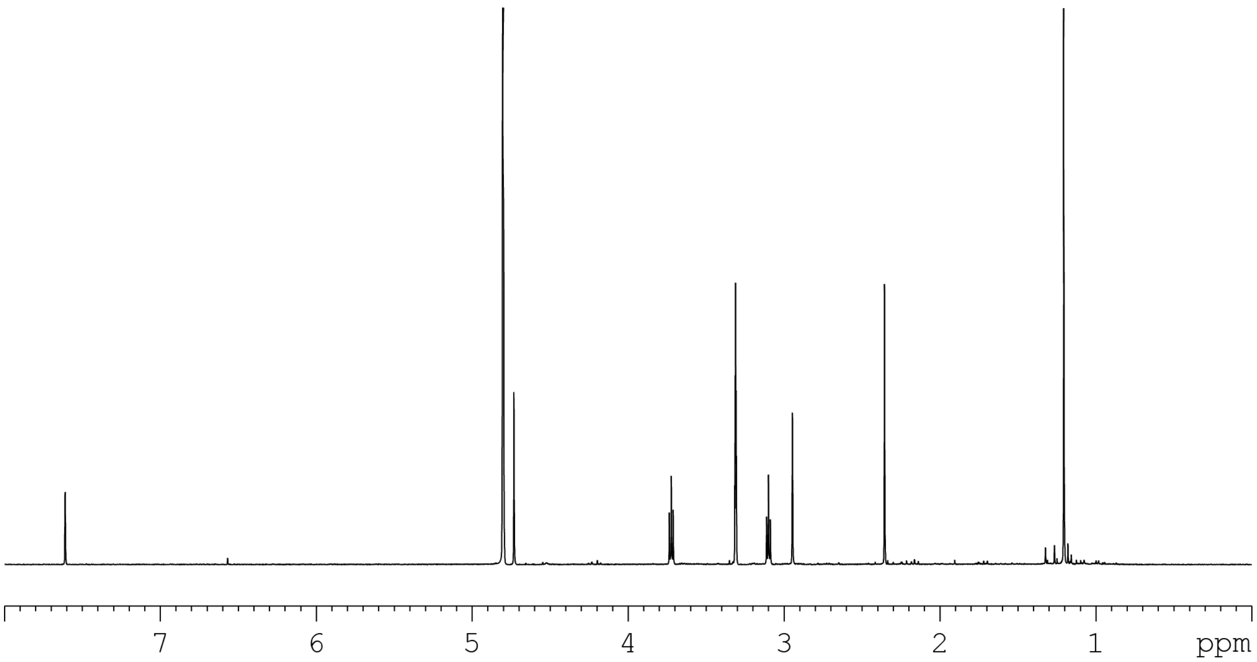

Figure S4. ^{13}C-NMR (methanol-d_4, 100 MHz) spectrum of **2**.

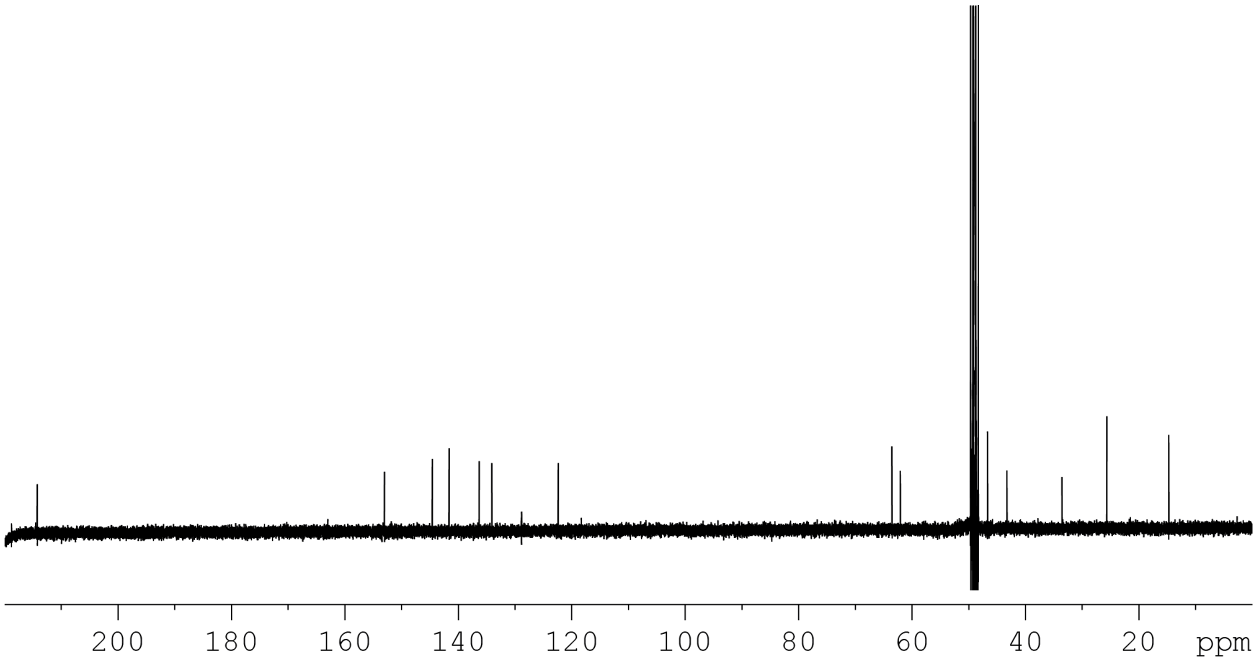

Figure S5. ^{1}H-NMR (methanol-d_4, 600 MHz) spectrum of **3**.

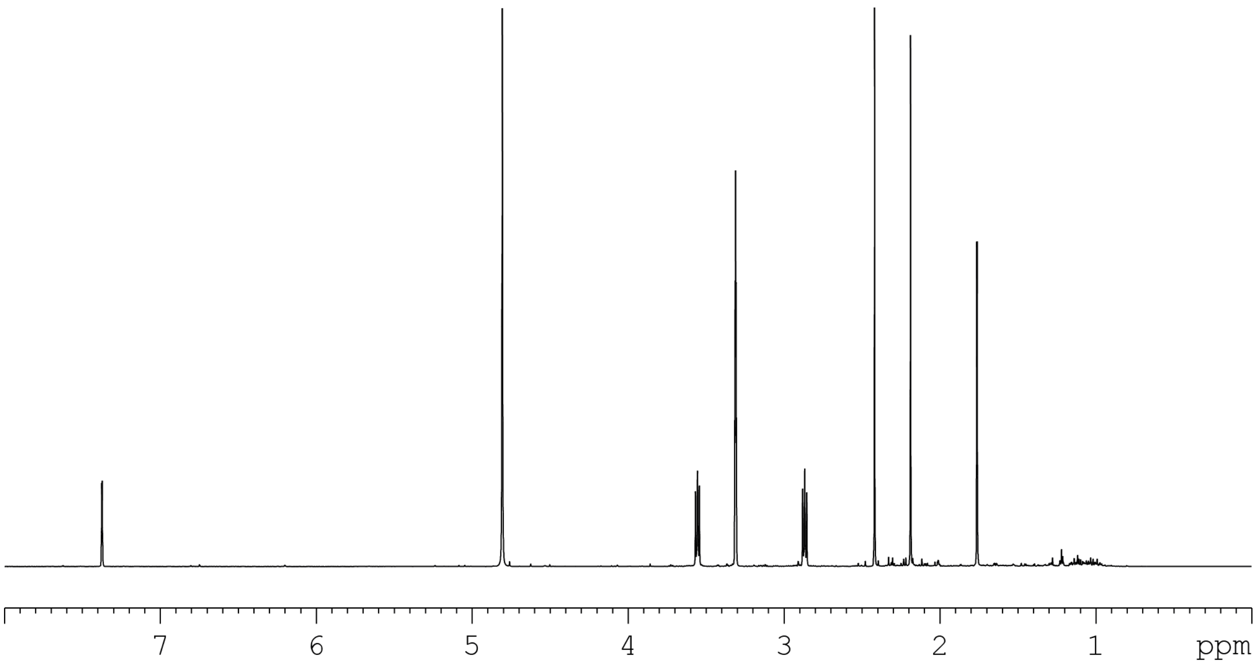

Figure S6. ^{13}C-NMR (methanol-d_4, 100 MHz) spectrum of **3**.

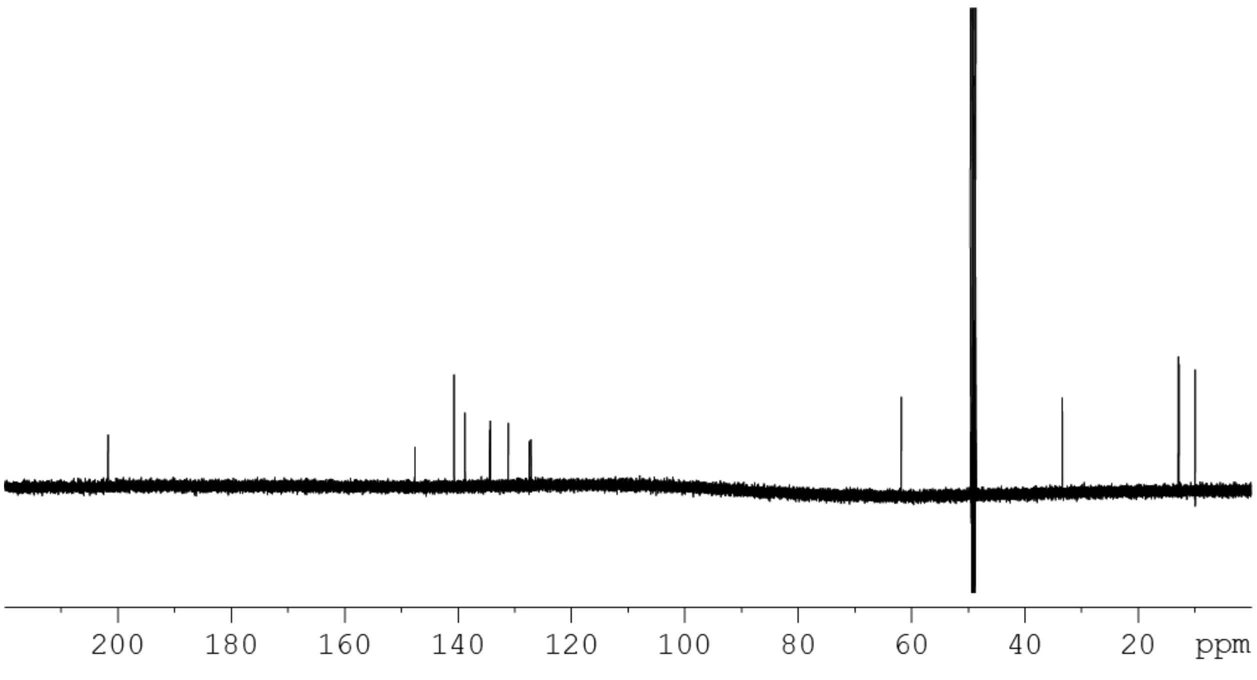

Figure S7. ^{1}H-NMR (methanol-d_4, 600 MHz) spectrum of pterosin M.

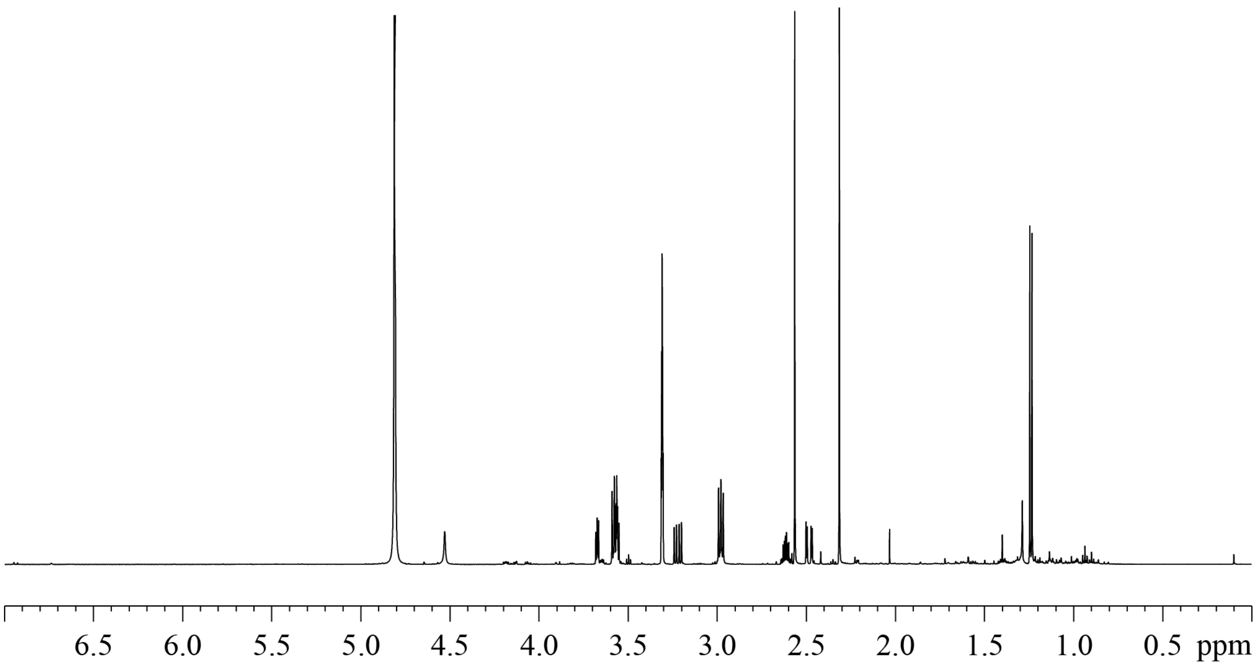

Figure S8. ^{13}C-NMR (methanol-d_4, 100 MHz) spectrum of pterosin M.

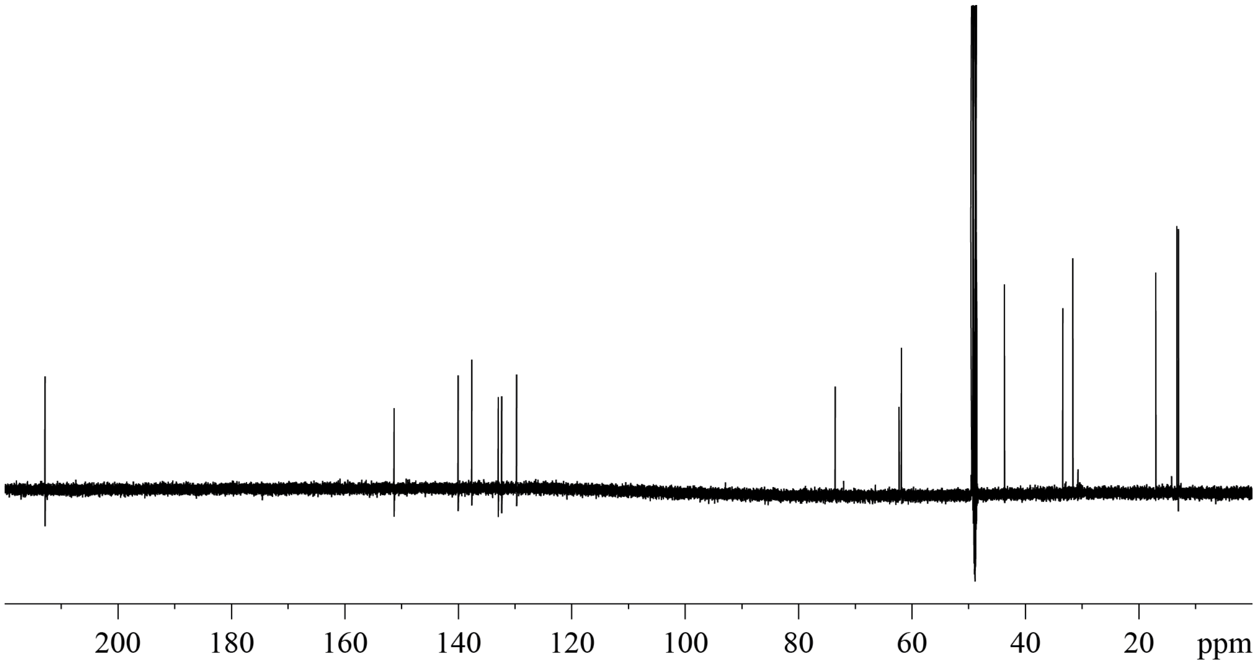

Figure S9. ^{1}H-NMR (methanol-d_4, 600 MHz) spectrum of echinolactone D.

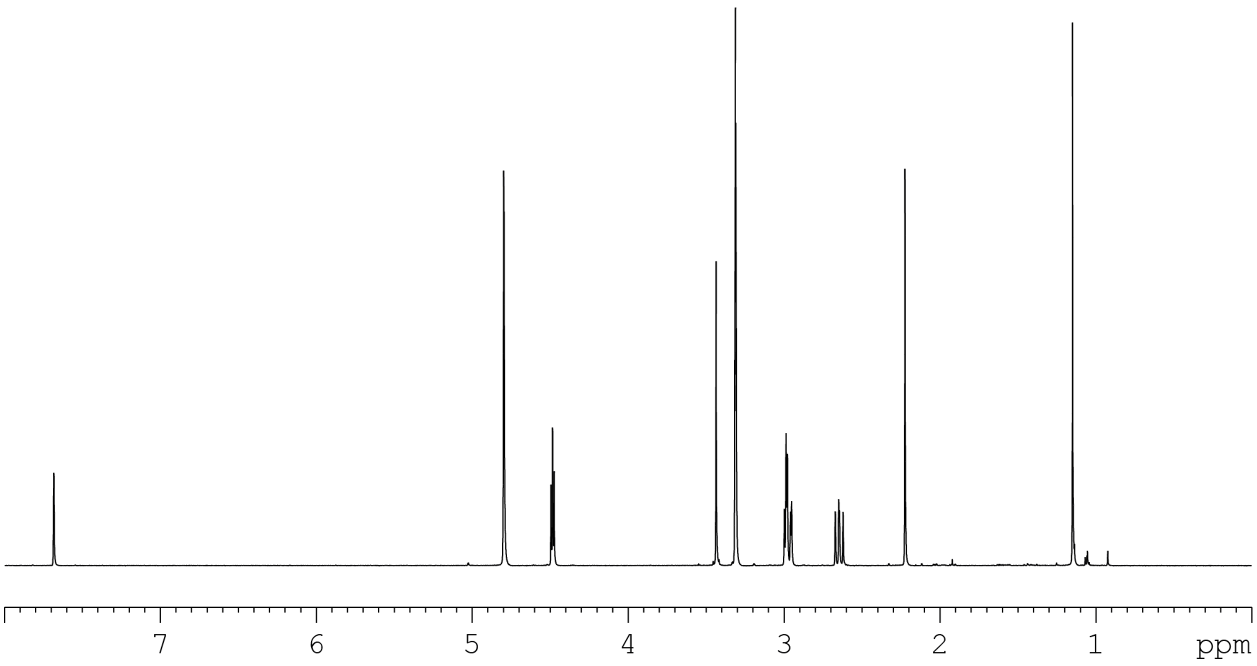

Figure S10. ^{13}C-NMR (methanol-d_4, 100 MHz) spectrum of echinolactone D.

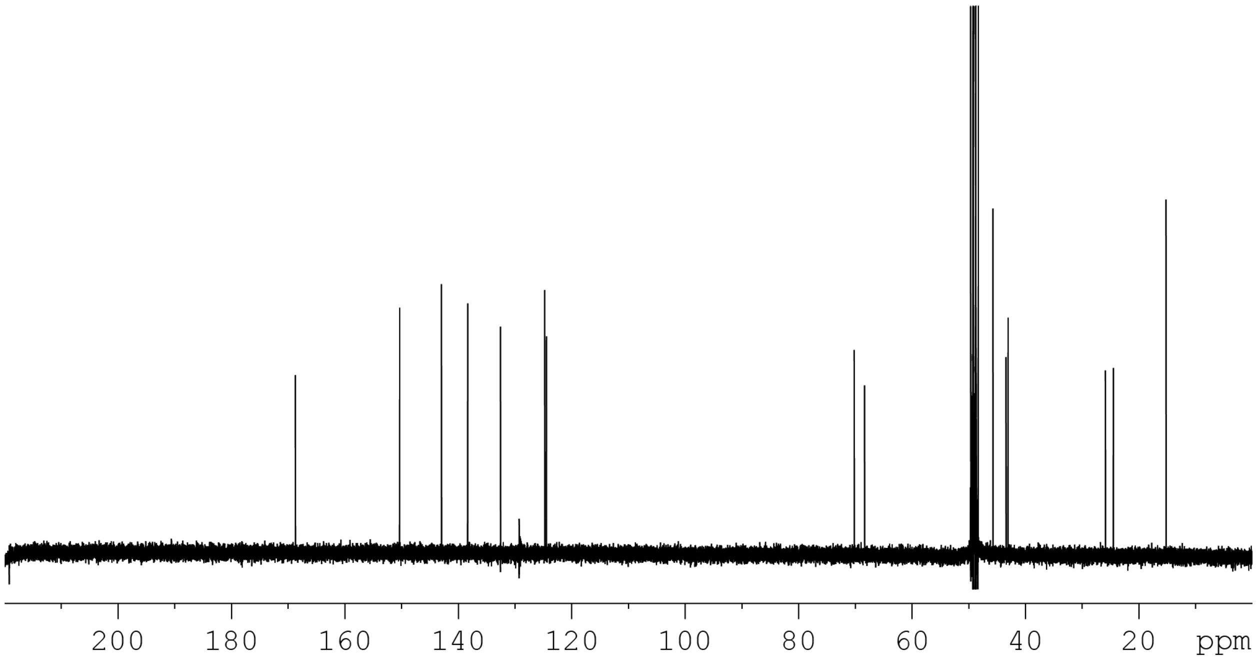

Figure S11. ^{1}H-NMR (methanol-d_4, 600 MHz) spectrum of radulactone.

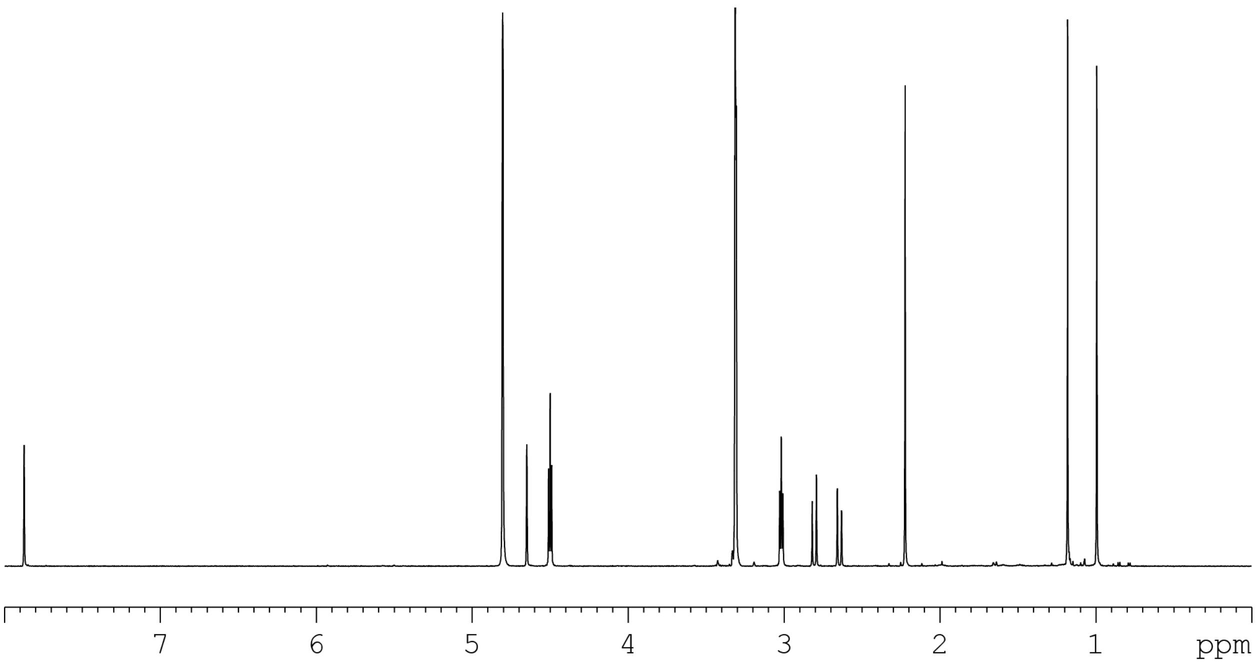

Figure S12. ^{13}C-NMR (methanol-d_4, 100 MHz) spectrum of radulactone.

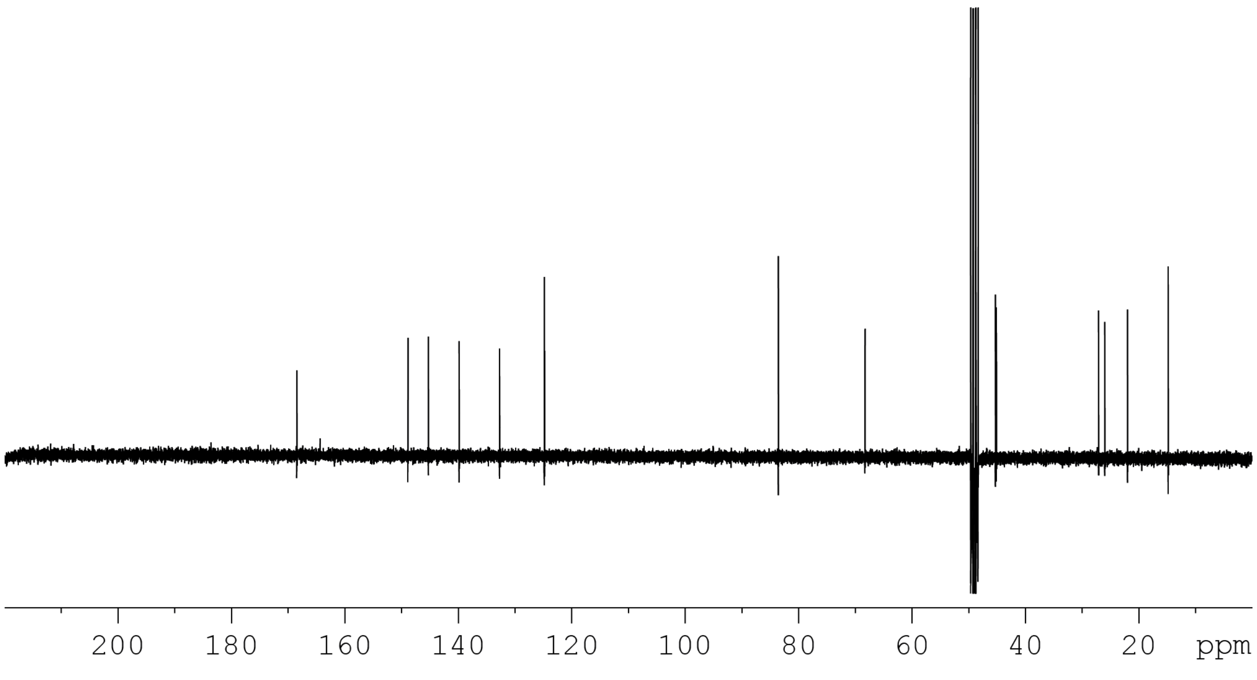

Figure S13. ^{1}H-NMR (methanol-d_4, 600 MHz) spectrum of echinolactone A.

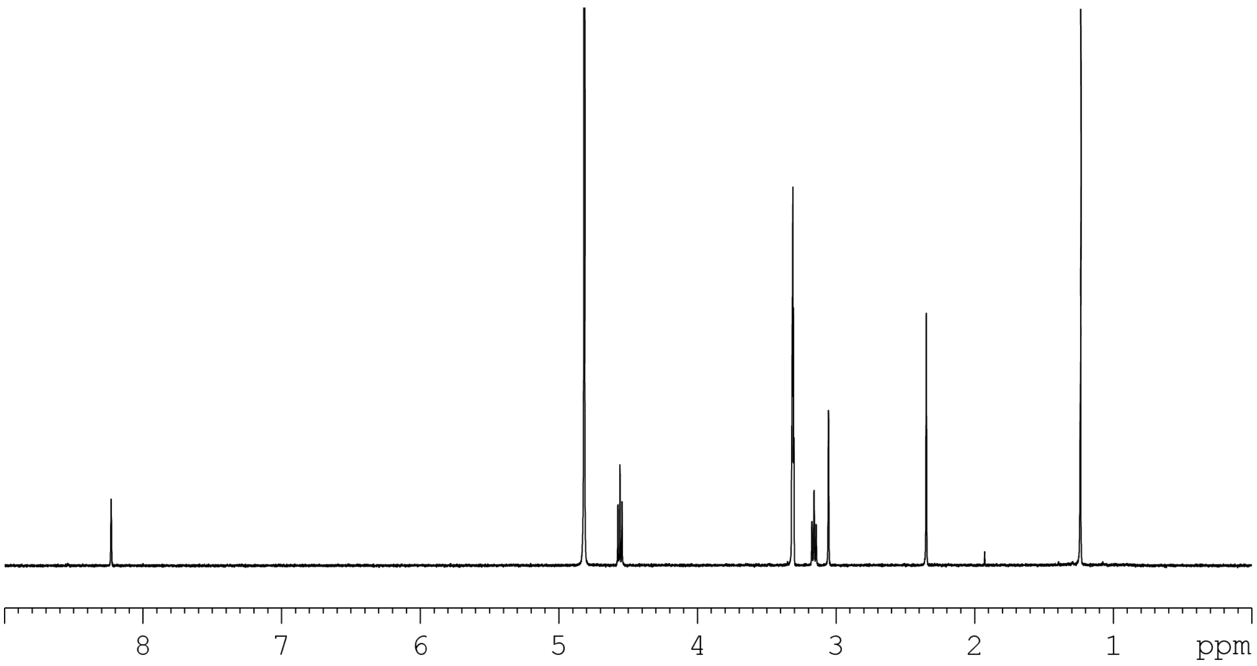

Figure S14. ^{13}C-NMR (methanol-d_4, 100 MHz) spectrum of echinolactone D.

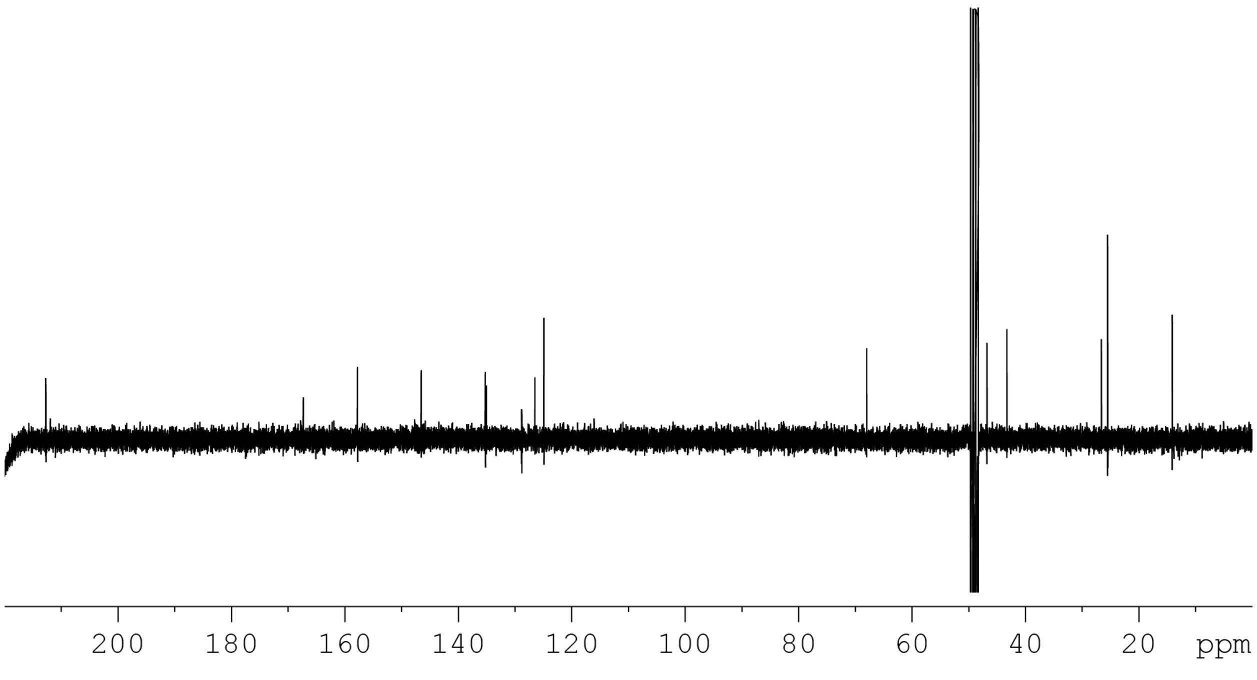

Figure S15. ^{1}H-NMR (acetone-d_6, 600 MHz) spectrum of **1a**.

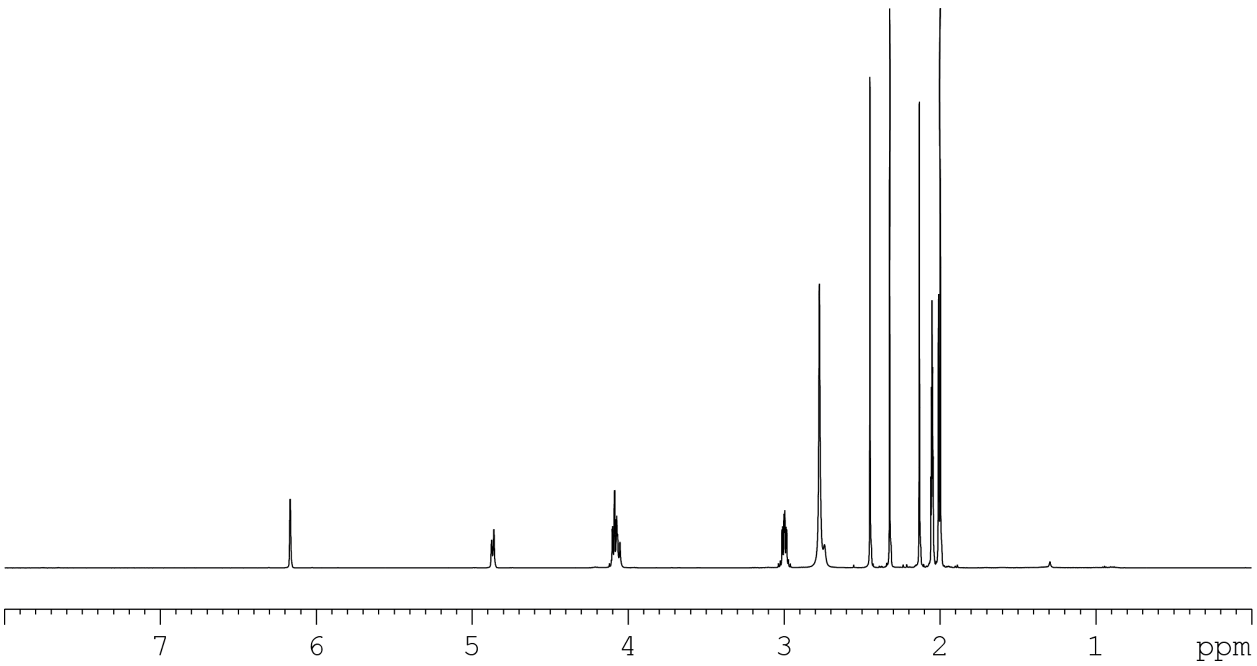

Figure S16. ^{1}H-NMR (acetone-d_6, 600 MHz) spectrum of the (S)-MTPA ester of **1a**.

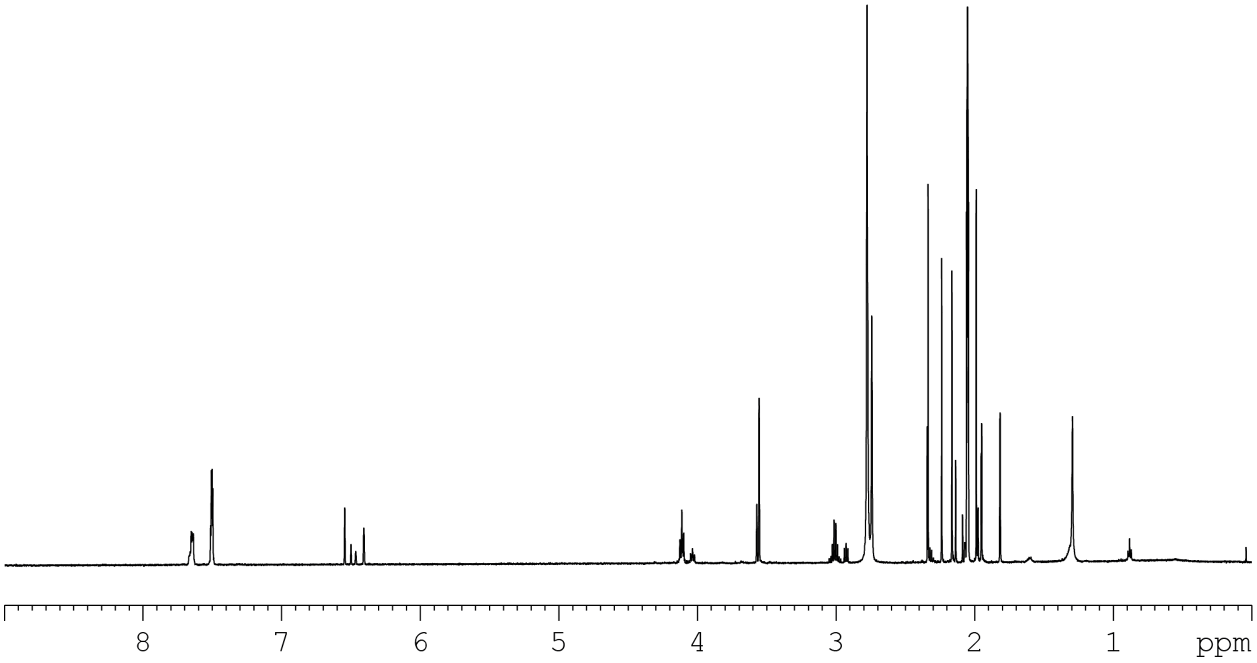

Figure S17. ^{1}H-NMR (acetone-d_6, 600 MHz) spectrum of the (R)-MTPA ester of **1a**.

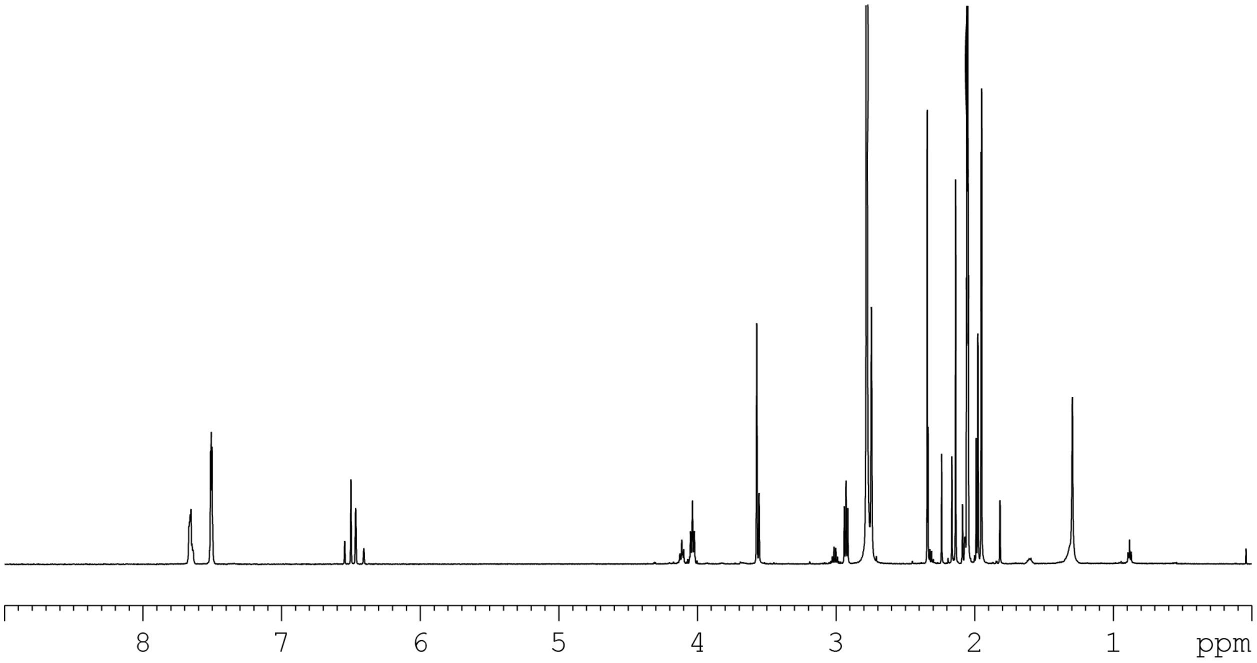

Figure S18. ^{1}H-NMR (methanol-d_6, 600 MHz) spectrum of the reaction product of **3** and cysteine.

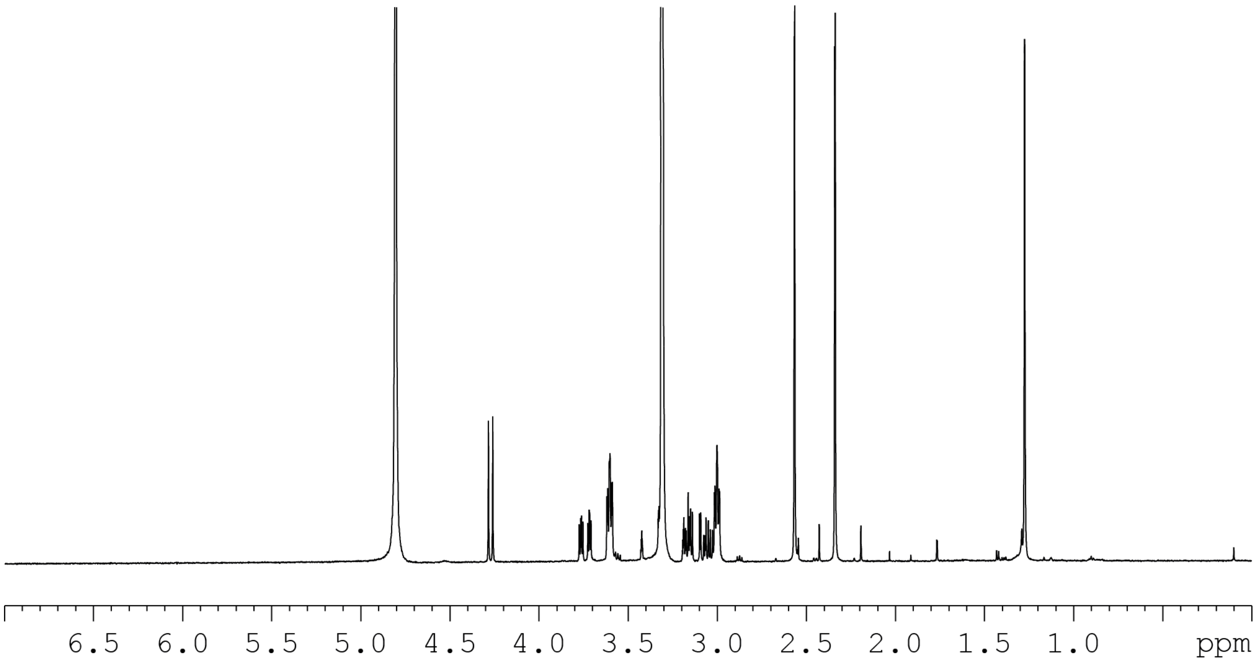

Figure S19. Structure of **1a** (R = OH) and the R- and S-MTPA monoesters of **1a** (R = R-MTPA and R = S-MTPA, respectively).

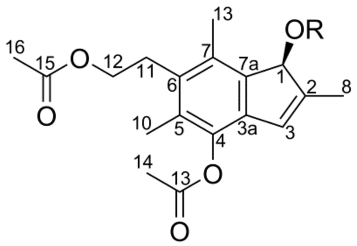

Table S1. ^{1}H-NMR data for compound **1a** and the R- and S-MTPA monoesters of **1a** (acetone-d_6, 30 °C, 600 MHz).

pos.	1a	R-MTPA Ester of 1a	S-MTPA Ester of 1a
	δ_H (*J* in Hz)	δ_H (*J* in Hz)	δ_H (*J* in Hz)
**1**	4.87, m	6.47, s	6.54, s
**2**			
**3**	6.17, bs	6.5, s	6.41, s
**4**			
**4a**			
**5**			
**6**			
**7**			
**7a**			
**8**	2.01, bs	1.98, bs	1.82, s
**9**			
**10**	2.13, s	2.14, s	2.16, s
**11**	3.00, m	2.93, m	3.01, m
**12**	4.09, m	4.00, m	4.11, m
**13**	2.45, s	1,95, s	2.23, s
**14**	2.32, s	2.34, s	2.34, s
**15**			
**16**	2.00, s	1.95, s	1.99, s
